# Synergistic population coding of natural communication stimuli by hindbrain electrosensory neurons

**DOI:** 10.1038/s41598-021-90413-1

**Published:** 2021-05-25

**Authors:** Ziqi Wang, Maurice J. Chacron

**Affiliations:** Department of Physiology, McGill University, Montreal, Canada

**Keywords:** Neuroscience, Sensory processing

## Abstract

Understanding how neural populations encode natural stimuli with complex spatiotemporal structure to give rise to perception remains a central problem in neuroscience. Here we investigated population coding of natural communication stimuli by hindbrain neurons within the electrosensory system of weakly electric fish *Apteronotus leptorhynchus*. Overall, we found that simultaneously recorded neural activities were correlated: signal but not noise correlations were variable depending on the stimulus waveform as well as the distance between neurons. Combining the neural activities using an equal-weight sum gave rise to discrimination performance between different stimulus waveforms that was limited by redundancy introduced by noise correlations. However, using an evolutionary algorithm to assign different weights to individual neurons before combining their activities (i.e., a weighted sum) gave rise to increased discrimination performance by revealing synergistic interactions between neural activities. Our results thus demonstrate that correlations between the neural activities of hindbrain electrosensory neurons can enhance information about the structure of natural communication stimuli that allow for reliable discrimination between different waveforms by downstream brain areas.

## Introduction

How neural populations encode sensory information (i.e., population coding) is one of the most intriguing questions in neuroscience and has been extensively studied^1–5^. Understanding population coding is complicated by the fact that neural activities are not independent of each other but are instead correlated^2,4,6,7^. The effects of correlations on coding remains under debate. Of particular interest are correlations between the trial-to-trial variability of neural activities (i.e. “noise correlations”) in relation to correlations between the mean neural responses to a given stimulus (i.e., “signal correlations”)^2^. While noise correlations can limit information transmission by introducing redundancy^3,8^, they can also introduce synergy and be beneficial to coding^9–11^. However, as most previous studies of population coding used artificial stimuli, the encoding of natural stimuli which often display complex spatiotemporal characteristics is less understood^12,13^.

Here, we studied population coding of natural communication stimuli in the wave-type weakly electric fish *Apteronotus leptorhynchus*. These fish emit a quasi-sinusoidal electric field through the electric organ discharge (EOD) and can sense field perturbations to locate preys as well as communicate with conspecifics^14,15^. These perturbations are detected by electroreceptors on the animals’ skin, which synapse onto pyramidal cells within the electrosensory lateral line lobe (ELL). ELL pyramidal cells can be categorized into ON cells and OFF cells, which respond to increases and decreases of the EOD amplitude, respectively. Pyramidal cells constitute the main output neurons of the ELL and project directly to the midbrain area *torus semicircularis*, and indirectly to higher brain areas that generate perception and behavior^16^. During social interactions, weakly electric fish communicate using brief changes in their EOD called “chirps” whose attributes vary over a wide range and thus give rise to very heterogeneous stimulus waveforms^17–22^. Previous studies have primarily focused on understanding how single electrosensory neurons respond to chirp stimuli^23,24^ and used these recordings to study population coding^25,26^. However, a limitation is that, because the neural recordings were not performed simultaneously, the effects of noise correlations were not considered. Importantly, ELL pyramidal cells display correlations between their activities in the absence of stimulation^27^, which tend to give rise to noise correlations during stimulation^28,29^. Here, to investigate how correlations affect population coding of chirps by ELL pyramidal cell populations, we used multi-channel Neuropixels probes to record simultaneously the activities of multiple ELL pyramidal cells in response to chirp stimuli.

## Results

**Figure 1:**
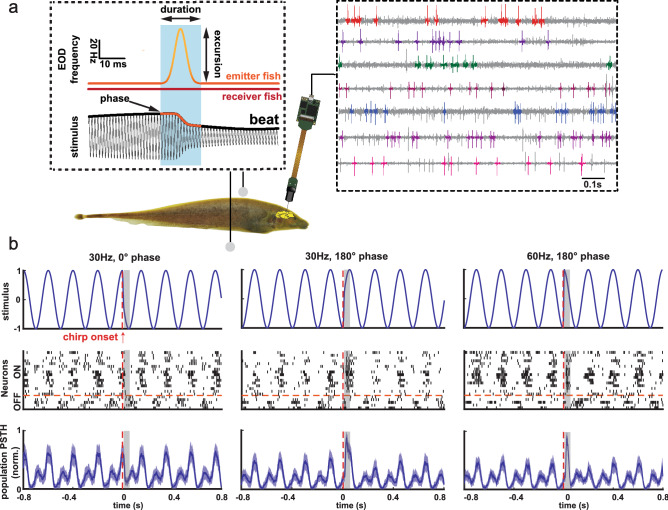
Neuropixels probes were used to record extracellular activities of ELL pyramidal cells responding to chirps created using adobe illustrator CS6 v 16.0 (www.adobe.com). (**a**) Left: schematics demonstrating chirps stimuli used in the experiments and experimental set up. Right: recorded activities from example channels using Neuropixels probes with spikes of different neurons highlighted in colors. (**b**) Left: stimulus waveform (top) consisting of a 5 Hz beat with a chirp (vertical red dashed line), raster plot of ON and OFF cells (middle), and the mean and standard deviation (shaded areas) of normalized population PSTHs across different trials (see “Materials and methods”) (bottom) for chirp with 30 Hz excursion frequency at 0° of beat phase. The grey rectangle indicates the 40 ms chirp evaluation time window. Middle: same plots for chirp with 30 Hz excursion frequency at 180° of beat phase. Right: same plots for chirp with 60 Hz excursion frequency at 180° of beat phase.

Here we investigated how ELL pyramidal cell populations encode chirps with different attributes. During social interaction, interference between the EODs of two fish form a beat (i.e., a sinusoidal modulation in EOD amplitude; Fig. 1a, top left). Chirps consist of transient increases in the EOD frequency of one fish (i.e., the emitter fish) and will give rise to a transient modulation of the beat waveform as sensed by the receiver fish. Differences in the duration of the frequency increase, its excursion, and the beat phase at which the chirp occurs will thus give rise to different stimulus waveforms^30^. Figure 1b shows three example chirp stimulus waveforms (top) as well as raster plots of ON and OFF cells (middle) and population peri-stimulus time histograms (population PSTHs; bottom) in response to each stimulus. We recorded the activities of multiple ELL pyramidal cells simultaneously using Neuropixels probes (Fig. 1a, right) in response to chirp stimuli that were delivered to the fish through a pair of electrodes located on either side of the fish (Fig. 1a, bottom left). We considered responses to chirps within a 40 ms time window that started 8 ms after chirp onset to account for transmission delays (see “Materials and methods”).

### Signal but not noise correlations vary with distance and stimuli

**Figure 2:**
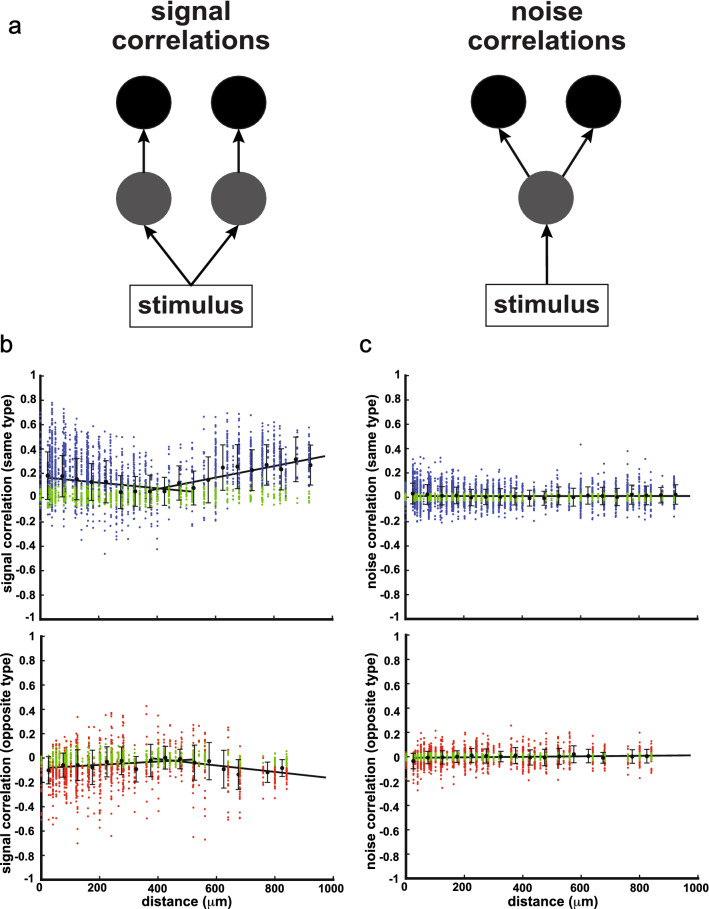
Signal but not noise correlations varied with distance. (**a**) Schematics showing how signal and noise correlations arise created using adobe illustrator CS6 v 16.0 (www.adobe.com). While signal correlation arises from similarity in mean responses to stimuli (left), noise correlation instead arises from shared noisy synaptic inputs (right). (**b**) Top: signal correlations of same-type pairs (i.e., pairs of either ON or OFF cells) as a function of distance (blue dots). Distance was discretized into 20 bins (50 microns per bin) and signal correlations for pairs that fall within the same bin were averaged (black dots, error bars indicate standard deviation). Signal correlations first decreased and then increased with distance (from 0 to 550 microns: linear regression, r = − 0.74, p = 0.011; from 400 to 1000 microns: linear regression, r = 0.91, p = 4.2 × 10^–5^). Bottom: signal correlations of opposite-type pairs (i.e., pairs containing one ON and one OFF cell) as a function of distance (red dots). Signal correlations first increased and then decreased with distance when the data was averaged within bins (from 0 to 550 microns: linear regression, r = 0.66, p = 0.030; from 400 to 1000 microns: linear regression, r = − 0.81, p = 8.3 × 10^–3^). We note that qualitatively similar results were obtained when performing a linear regression on the data without averaging (same type: from 0 to 550 microns: linear regression, r = − 0.26, p = 4.7 × 10^–37^; from 400 to 1000 microns: linear regression, r = 0.34, p = 2.8 × 10^–26^; opposite type: from 0 to 550 microns: linear regression, r = 0.18, p = 2.2 × 10^–13^; from 400 to 1000 microns: linear regression, r = − 0.32, p = 2.0 × 10^–14^). (**c**) Top: same as (**b**), but for noise correlations. There was no significant correlation between noise correlations and distance for both same-type pairs and opposite-type pairs (same-type pairs: linear regression, r = 0.020, p = 0.96; opposite type pairs: linear regression, r = 0.42, p = 0.10). When performing a linear regression on the data without averaging, we found a negligible but significant relationship between noise correlations and distance both for same type pairs (slope = − 1.3 × 10^–5^, r = − 0.045, p = 0.014) and for opposite type pairs (slope = 2.5 × 10^–5^, r = 0.096, p = 2.2 × 10^–5^). However, note that the slopes are infinitesimally small in magnitude in both cases. In panels b and c, correlation coefficient values that were deemed non-significant at the p = 0.05 level using the function “corrcoeff” in Matlab are plotted in green.

As previous studies have shown that the correlation structure (i.e., the relationship between signal and noise correlations) strongly impacts population coding^2^, including in ELL pyramidal cells but for stimuli other than those considered here^28,31^, we first investigated signal and noise correlations between ELL pyramidal cell pairs during chirp stimulation. Signal correlations represent similarities between the mean responses of two neurons to a given stimulus (Fig. 2a, left), while noise correlations are instead correlations between the trial-to-trial variabilities of neural responses to repeated presentations of a given stimulus and arise due to shared noisy synaptic input (Fig. 2a, right).

We found that ELL pyramidal cells displayed both signal and noise correlations in their activities in response to chirp stimuli. Specifically, signal correlations of same-type (i.e., pairs containing either ON cells or OFF cells) and opposite-type pairs (i.e., pairs containing both ON and OFF cells) were on average positive and negative respectively (Fig. 2b, compare top and bottom panels). In contrast, noise correlations were similarly distributed around 0 for both same-type and opposite-type pairs (Fig. 2c, compare top and bottom panels). Interestingly, for same-type pairs, signal correlations first decreased and then increased with increasing distance between the probe sites on which both neurons were recorded (Fig. 2b top, from 0 to 550 μm: linear regression, r = − 0.74, p = 0.011; from 400 to 1000 μm: linear regression, r = 0.91, p = 4.2 × 10^–5^). For opposite-type pairs, the opposite trend was observed in that signal correlation first increased and then decreased with increasing distance (Fig. 2b bottom, from 0 to 550 μm: linear regression, r = 0.66, p = 0.030; from 400 to 1000 μm: linear regression, r = -0.81, p = 8.3 × 10^–3^). However, noise correlations were largely independent of distance for both same-type and opposite-type pairs (Fig. 2c, same-type pairs: linear regression, r = 0.020, p = 0.96; opposite type pairs: linear regression, r = 0.42, p = 0.10).

**Figure 3:**
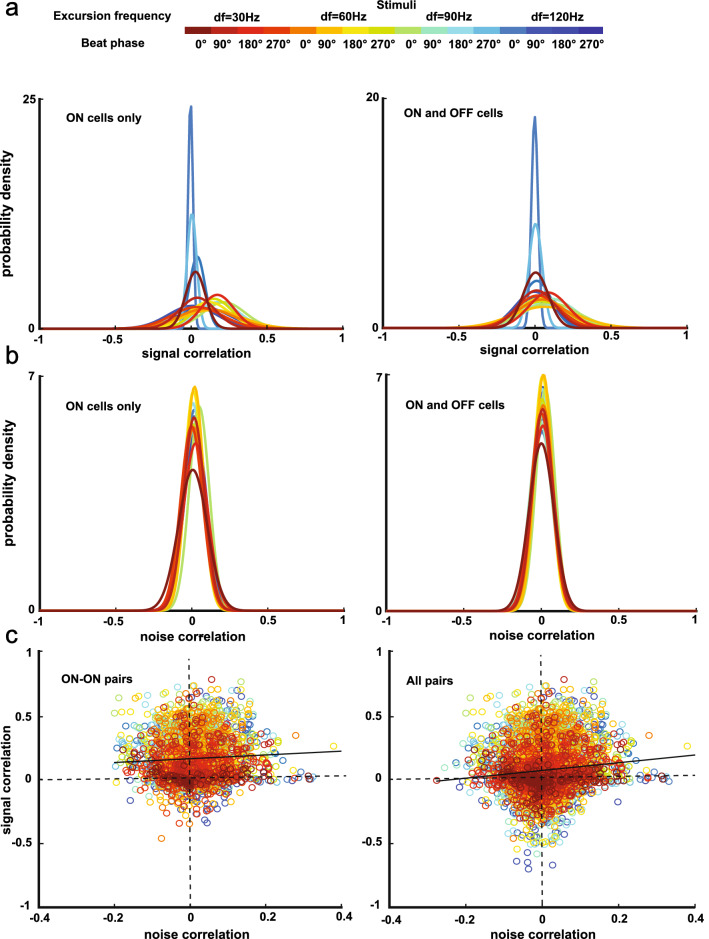
Correlation structure during chirp stimulation. (**a**) The probability distribution of signal correlations for each chirp stimulus waveform used. There were significant differences between the distributions for the population with only ON cells (left) and the population with both ON and OFF cells (right) (ON cells only: Friedman's test, p = 4.0 × 10^–44^; ON and OFF cells: Friedman’s test, p = 1.1 × 10^–16^). (**b**) Same as (**a**) but for noise correlations. There were significant differences between the distributions for the population with ON cells only (Friedman’s test, p = 0.020), while there were no significant differences between the distributions for the population with both ON and OFF cells (Friedman’s test, p = 0.17). (**c**) Noise correlations of ON-ON pairs only (left) and of all pairs (right) plotted against signal correlations of the same pairs. There were positive relationships between signal and noise correlations for ON–ON pairs and for all pairs (ON–ON pairs: linear regression, r = 0.060, p = 1.3 × 10^–3^; all pairs: linear regression, r = 0.11, p = 6.0 × 10^–15^).

Next, we looked at whether and, if so, how signal and noise correlations varied as a function of the different chirp stimulus waveforms used in this study. We found that for the population with only ON cells, the distributions of signal and noise correlations were significantly different from one another for different chirps (Fig. 3a left, Friedman’s test, p = 4.0 × 10^–44^; Fig. 3b left, Friedman’s test, p = 0.020). However, for the population with both ON and OFF cells, while the distributions of signal correlation were significantly different (Fig. 3a right, Friedman’s test, p = 1.1 × 10^–16^), noise correlation distributions did not change significantly (Fig. 3b right, Friedman’s test, p = 0.17). Furthermore, we noticed that noise and signal correlations were not independent of each other. The signal and noise correlations for both ON-ON pairs and for all pairs are shown in Fig. 3c. Overall, there were positive but weak correlations between signal and noise correlations for both cases (Fig. 3c left, linear regression, r = 0.060, p = 1.3 × 10^–3^; Fig. 3c right, linear regression, r = 0.11, p = 6.0 × 10^–15^). Thus, our results at this stage show that, while signal correlations were strongly dependent on distance and chirp stimulus waveform, this was generally not the case for noise correlations.

### Decoding ELL pyramidal cells activities with equal-weight sum and weighted sum

We next quantified the performance of a classifier at correctly discriminating between neural responses generated by a given chirp stimulus waveforms (see “Materials and methods”). In short, neural activities of all neurons were combined in different manners to obtain the population activity. The population activities obtained in response to different chirp waveforms were then compared across different stimulus trials using the van Rossum metric^32^. Thus, a given population activity was assigned as being generated by a certain stimulus *i* if the distance between this activity and the chosen template for stimulus *i* was lower than all other distances computed using chosen templates for other stimuli (see “Materials and methods”). In practice, the trial-averaged population activities were chosen as templates. The performance of the classifier is represented by a confusion matrix where each entry (*i*,*j*) is the probability that a response which was actually generated by stimulus *i* is classified as generated by stimulus *j*. As such, the diagonal elements of the confusion matrix give the amount of correct classification whereas the off-diagonal elements instead give the amount of incorrect classification.

**Figure 4:**
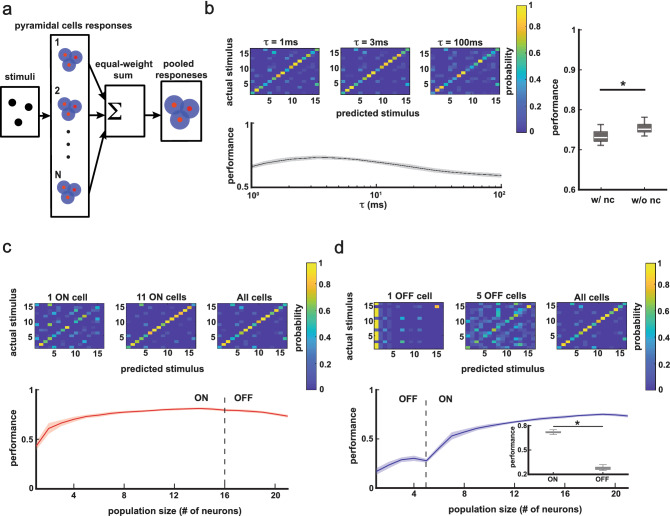
Discrimination performances of population activities when using an equal-weight sum to combine neural activities. (**a**) Schematics showing how the responses of ELL pyramidal cells were summed with equal weights. (**b**) Left top: confusion matrices where each entry is the probability of a stimulus i predicted as stimulus j (prediction based on distance between neural responses quantified by van Rossum metric with timescale τ, see “Materials and methods” for details) for a population of 21 neurons consisting of 16 ON cells and 5 OFF cells with τ = 1, 3 and 100 ms. Left bottom: discrimination performance as a function of τ. The shaded areas represent standard deviation when using 30 different sub-trials (60% of all trials), and same for (**c**) and (**d**). The range of τ values for which performance was higher than 90% of the maximum is 6 ms. Right: boxplots showing that equal-weight sum of neural activities without noise correlations (right) had better performance than that with noise correlations (left; one-way ANOVA, p = 1.3 × 10^–4^). (**c**) The effect of population size on discrimination performance. ON cells were first considered before OFF cells. Top: confusion matrices for populations of 1 ON cell, 11 ON cells, and all cells (16 ON cells and 5 OFF cells) with τ = 3 ms. Bottom: discrimination performance as a function of population size. (**d**) Same as (**c**) but OFF cells were first considered before ON cells. Top: confusion matrices for populations of 1 OFF cell, 5 OFF cells, and all cells (16 ON cells and 5 OFF cells) with τ = 3 ms. Bottom: discrimination performance as a function of population size. Inset: boxplot showing that 5 ON cells had better performance than 5 OFF cells (one-way ANOVA, p = 1.0 × 10^–66^).

First, we combined the neural activities by performing a linear sum giving the same weight to each neuron (Fig. 4a). To quantify the effects of noise correlations, the performance of the classifier was evaluated on the neural responses as well as neural responses that were randomly shuffled with respect to trial order (see “Materials and methods”). Performances obtained with and without noise correlations were significantly above chance level (with noise correlations, one-sample t-test, p = 3.9 × 10^–50^; without noise correlations, one-sample t-test, p = 1.5 × 10^–54^). We quantified the effect of timescale of encoding used in the van Rossum metric on the performance. This is important as small timescales put more emphasis on precise spike timing whereas larger timescales instead place more emphasis on slower variations in the firing rate^32^. We found that maximal performance was observed using a timescale of ~ 3 ms (Fig. 4b left), indicating that precise spike timing can be used to reliably discriminate between different chirp stimulus waveforms. The performance when noise correlations were removed was higher than that obtained for the raw data (Fig. 4b right, one-way ANOVA, p = 1.3 × 10^–4^), indicating that noise correlations have a detrimental effect on discrimination performance. Next, we analyzed how discrimination performance varied as a function of population size. We separated the entire population into ON cells and OFF cells and increased the population size by adding either ON cells or OFF cells first. We found that when increasing population size by first adding the ON cells, the performance increased when ON cells only were first considered and actually decreased when OFF cells were added to the pool (Fig. 4c). Interestingly, when increasing population size by first adding the OFF cells, the performance started with low values and increased slowly, but later increased drastically when ON cells were added (Fig. 4d). We found that ON cell populations had much better performance than OFF cell populations (Fig. 4d inset, one-way ANOVA, p = 1.0 × 10^–66^). These results were consistent with the previous findings that single ON cells instead of single OFF cells better respond to chirps^33^.

**Figure 5:**
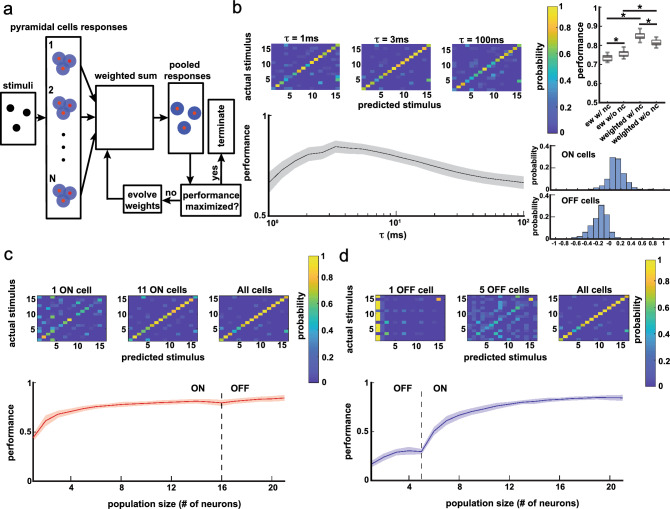
Discrimination performances of population activities when taking the weighted population sum. (**a**) Schematics showing how the responses of ELL pyramidal cells were summed with different weights assigned for different neurons. The weights were generated by an evolutionary algorithm. If the weights generated gave a better performance, they replaced the previous weights; if the weights generated did not improve the performance for 10 iterations (performance maximized), the evolutionary algorithm was terminated (see “Materials and methods” for details). (**b**) Left top: confusion matrices where each entry is the probability of a stimulus i predicted as stimulus j (prediction based on the distance between neural responses quantified by van Rossum metric with timescale τ, see “Materials and methods” for details) for a population of 21 neurons consisting of 16 ON and 5 OFF cells with τ = 1, 3 and 100 ms. Left bottom: discrimination performance as a function of τ. The shaded areas represent standard deviation of performance from different simulations of the evolutionary algorithm (30 in total), and same for (**c**) and (**d**). The range of τ values for which performance was higher than 90% of the maximum is 5.3 ms, which is similar to that obtained in the equal-weighted case (Fig. 4b). Right top: boxplots showing that weighted sums of neural activities improved performance for both with and without noise correlations (with noise correlations, one-way ANOVA, p = 1.1 × 10^–39^; without noise correlations, one-way ANOVA, p = 8.7 × 10^–19^); also, equal-weight sum of neural activities without noise correlations had better performance than those with noise correlations (one-way ANOVA, p = 1.3 × 10^–4^), while weighted sum of neural activities with noise correlations had better performance than those without noise correlations (one-way ANOVA, p = 5.3 × 10^–19^). Right bottom: the probability distributions of weights assigned to ON cells and OFF cells over 30 runs of the evolutionary algorithm. (**c**) The effect of population size on discrimination performance. ON cells were first considered before OFF cells. Top: confusion matrices for populations of 1 ON cell, 11 ON cells, and all cells (16 ON cells and 5 OFF cells) with τ = 3 ms. Bottom: discrimination performance as a function of population size. (**d**) Same as (**c**) but now OFF cells were first considered before ON cells. Top: confusion matrices for populations of 1 OFF cell, 5 OFF cells, and all cells (16 ON cells and 5 OFF cells) with τ = 3 ms. Bottom: discrimination performance as a function of population size.

Next, we combined the neural activities of all neurons using a weighted sum (i.e., a sum with unequal weights) (Fig. 5a). To find the weights that give rise to the best discrimination performance, we used an evolutionary algorithm (see “Materials and methods”; Fig. 5a). The effect of timescale of encoding on performance was similar as in the case of the equal-weight sum (Fig. 5b left). Overall, the performance improved significantly when performing a weighted sum as compared to that obtained with equal-weight sum with and without noise correlations (Fig. 5b right top, equal-weight with noise correlations vs. weighted with noise correlations, one-way ANOVA, p = 1.1 × 10^–39^; equal-weight without noise correlations vs. weighted without noise correlations, one-way ANOVA, p = 8.7 × 10^–19^). Weight distributions for ON and OFF cells were largely mirror images of one-another (Fig. 5b right bottom, ON cells: mean = 0.12, std = 0.16; OFF cells: mean = − 0.19, std = 0.15) and were significantly different (two-sample Kolmogorov–Smirnov test, p = 9.0 × 10^–62^). Further, we noticed that, unlike the equal-weight case, noise correlations were actually beneficial as removing them significantly reduced performance (Fig. 5b right, weighted with noise correlations vs. weighted without noise correlations, one-way ANOVA, p = 5.3 × 10^–19^). For the effect of population size on performance, adding OFF cells to the ON cells population did not decrease the performance (Fig. 5c), in contrast to the equal-weight case (Fig. 4c); however, a population with only OFF cells still had a poor performance in the weighted case (Fig. 5d). It is important to note that population consisting of only ON cells displayed much better performance in the weighted case than in the equal-weighted case (compare Figs. 5c and 4c). As such, the improvement in performance is not due to considering both ON and OFF cells with opposite weights. Rather, such improvement is largely due to heterogeneities within the ON cell population.

**Figure 6:**
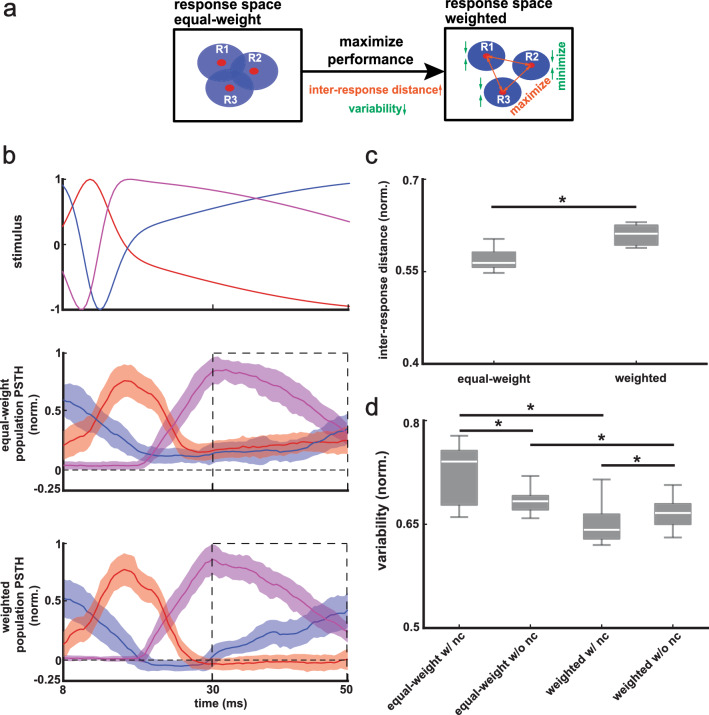
Weighted sums maximized the performance by increasing distances between trials-averaged responses to different stimuli and reducing trial-to-trial variability. (**a**) Schematics showing that weighted sums of neural activities maximize the performance by increasing the inter-response distance (i.e., distances between trials-averaged responses to different stimuli) and reducing response variability. These were created using adobe illustrator CS6 v 16.0 (www.adobe.com). (**b**) Top: three example chirp stimuli. The waveforms are shifted to the right by 8 ms to account for the common synaptic delay of chirp responses. Middle and bottom: the means and standard deviations (shaded areas) of normalized population PSTHs across different trials of the example chirp stimuli under equal-weight and weighted sum. Horizontal dashed line indicates zero. Dashed squares indicate that responses to different stimuli under weighted sum are more different from each other compared to responses under equal-weight sum. (**c**) Boxplots showing that weighted sums of neural activities had higher inter-response distance than equal-weight sums of neural activities (one-way ANOVA, p = 4.5 × 10^–4^). (**d**) Boxplots showing that weighted sums of neural activities had lower response variability than equal-weight sums of neural activities (with noise correlations, one-way ANOVA, p = 3.9 × 10^–29^; without noise correlations, one-way ANOVA, p = 3.4 × 10^–17^); also, equal-weight sums of neural activities without noise correlations had lower response variability than equal-weight sums with noise correlations (one-way ANOVA, p = 3.1 × 10^–9^) while weighted sums of neural activities without noise correlations had higher response variability than weighted sums with noise correlations (one-way ANOVA, p = 0.040).

Why is there an overall performance increase when using a weighted sum vs. an unweighted sum? Intuitively, increases in performance can occur when the set of responses elicited by different stimuli become more distant from one another and thus more discriminable. However, increases in performance can also occur if the size of these sets decreases (Fig. 6a). Figure 6b shows three example stimulus waveforms (left top panel) as well as population PSTHs when taking equal-weight (left middle panel) and weighted (left bottom panel) sums. It was seen that the population activities were more different from each other (see dashed rectangle) when taking weighted sums, partly because a weighted sum with both positive and negative weights can lead to negative population activities while population activities obtained with equal-weight sum can only be positive by definition. Quantification of the distance between responses (see “Materials and methods”) confirmed that greater values were obtained when considering weighted sums than equal-weight sums (Fig. 6c, one-way ANOVA, p = 4.5 × 10^–4^). We next tested whether weighted neural responses were less variable than their equal-weight counterparts. To do so, we quantified the variability in the response using both weighted and equal-weight sums, as well as before and after removing noise correlations (see “Materials and methods”). We found that weighted sums reduced overall variability of neural activities, both with and without noise correlations (Fig. 6d, equal-weight with noise correlations vs weighted with noise correlations, one-way ANOVA, p = 3.9 × 10^–29^; equal-weight without noise correlations vs weighted without noise correlations, one-way ANOVA, p = 3.4 × 10^–17^). We also noticed that removing noise correlations reduced overall variability in the equal-weight case and increased overall variability in the weighted case (Fig. 6d, equal-weight with noise correlations vs equal-weight without noise correlations, one-way ANOVA, p = 3.1 × 10^–9^; weighted with noise correlations vs weighted without noise correlations, one-way ANOVA, p = 0.040).

### Weighted sums of ELL pyramidal cells activities eliminate redundancy and introduce synergy

**Figure 7:**
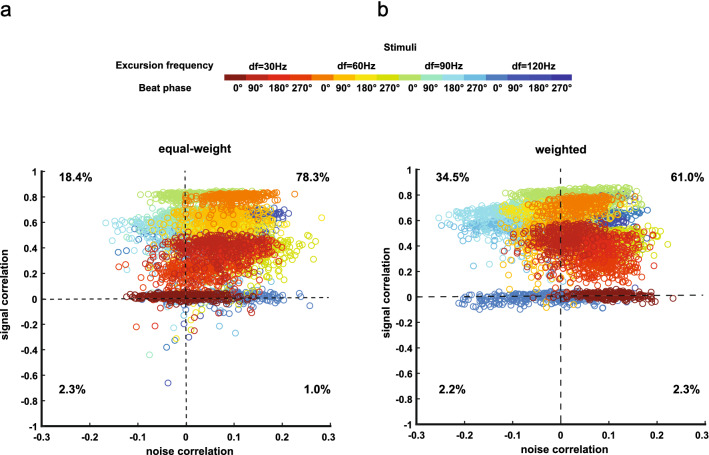
Weighted sums of neural activities eliminated redundancy and introduced synergy by yielding beneficial correlation structure. (**a**) Noise and signal correlations between two subpopulations, which were formed by partially summing the activities of entire population with equal weights (see “Materials and methods”). Different data points correspond to different bootstrap samples of partial sums. Percentages of data points in each quadrant are shown. (**b**) Same as (**a**) but the subpopulations were formed using weighted sums.

Why is the performance greater for weighted sums before removing noise correlations? Previous theoretical studies have shown that noise correlations can be beneficial to information transmission when their sign is opposite to that of signal correlations^2^. In order to study the correlation structures at a population level beyond two neurons, we combined the activities of subsets of neurons. Specifically, we divided our dataset into two subpopulations and considered correlations between the summed (either equal-weight or weighted) activities of both subpopulations^34^ (see “Materials and methods”). We found that, for equal-weight, signal and noise correlations were both predominantly positive (Fig. 7a, 78.3% of points in upper-right quadrant). However, this was much less the case for weighted sums, as more data points with signal and noise correlations having the opposite signs were observed (Fig. 7b, number of points in upper-left quadrant increased from 18.4 to 34.5%, while number of points in upper-right quadrant decreased from 78.3 to 61.0%). These findings thus confirm our hypothesis and explain why removing noise correlations led to lower performance when considering equal-weight sums but instead led to increased performance when considering weighted sums.

## Discussion

### Summary of results

In this study, we investigated for the first time how ELL pyramidal cell populations encode natural electro-communication stimuli by simultaneously recording the activities of multiple neurons. We first demonstrated that the activities of ELL pyramidal cells were correlated pairwise under chirp stimulation. Specifically, while signal correlations varied as a function of the physical distance between recording probe sites as well as stimulus waveform, noise correlations were instead largely independent of both distance and stimulus waveform. There was furthermore a positive relationship between signal and noise correlations. We next quantified the performance of a classifier at correctly discriminating which stimulus waveform was presented based on the combined neural activities of ELL pyramidal cells. When the activities were combined using an equal-weight sum, we found that ON cells have better discrimination performance than OFF cells with a combined (ON and OFF cells) correct discrimination performance around 75%. Noise correlations were overall detrimental to discrimination performance as their removal increased performance. When instead considering weighted sums and using an evolutionary algorithm to optimize the weights, we found increased performance up to 85%. Interestingly, noise correlations were then beneficial as removing them decreased performance. Further analysis revealed that the improved performance by weighted sum was the result of maximizing distance between trial-averaged responses to different chirp stimuli and minimizing overall variability. By considering correlations between the summed activities of subpopulations, we found that signal and noise correlations tended to have the same sign when considering equal-weight sums, which is detrimental to discrimination. In contrast, signal and noise correlations with opposite signs became relatively more dominant when considering weighted sums, which is beneficial to discrimination. Our results thus show that ELL pyramidal cells display significant correlations in their activities during chirp stimulation that can be either beneficial or detrimental to discriminability depending on how these activities are decoded by downstream brain areas.

### Origins of signal and noise correlations

Our results have shown that signal correlation magnitude first decreased with distance then increased. While the decrease can be explained by increasing dissimilarity in the receptive fields of neurons with increasing distance, the increase of signal correlations as the distance further increased is more puzzling. One possible explanation is that descending input from higher brain areas (i.e., feedback) modulate chirp responses to increase signal correlations. Indeed, ELL pyramidal cells receive abundant feedback consisting of both topographic and diffuse sources^35^. In particular, diffuse feedback was shown to affect signal correlations in ELL pyramidal cells to beat stimuli^29^ and enhance single neuron responses to chirps^36^. Such feedback originates from cerebellar granule cells, which make the ELL a cerebellum-like structure^37^. As such feedback originates from afferent input located far away from the cell within the non-classical receptive field^38,39^, we hypothesize that this might explain the increase in signal correlations observed for larger distances. Alternatively, the decrease and increase in signal correlations could be due to the fact that the recording probe went across different maps of ELL, from the lateral segment (LS) into the central lateral segment (CLS) thereby recording from cells in different segments that receive similar feedforward inputs from electroreceptor afferents. Further studies are needed to test these predictions.

In contrast, our results showed that noise correlations were invariant as the physical distance between neurons increased. These observations agree with previous findings in the visual cortex that noise correlations do not depend on the contact distance^40^. In general, noise correlations can arise from both bottom-up and top-down inputs as well as recurrent connections^41^. The amount of common input from electrosensory afferents to ELL pyramidal cells decreases as the distance between neurons increases^27,28^. Thus, if noise correlations were caused by common feedforward input, they would likely decay as distance between neurons increases. Therefore, it is likely that the descending input from cerebellar granule cells mentioned above strongly contribute to shaping noise correlations during chirp stimulation. Indeed, previous studies have shown that feedback can modulate noise correlations in response to beat stimuli^29^. The fact that a previous study of cerebellum found that parallel fibers can synchronize neural activities and no difference in correlations was found across pairs with different distance^42^ is consistent with our hypothesis.

### Optimized decoding of ELL pyramidal cells activities

Our results showed that a weighted sum of neural activities can improve discrimination performance, which was due in part to synergistic effects of noise correlations. These findings agreed with the previous studies showing that, rather than averaging neuronal responses by weighting them equally, weighting neurons differently can provide more information^43–45^. In this case, the weights were generated using an evolutionary algorithm to maximize the discrimination performance of electro-communication stimuli. We note that such “combinatorial codes” can recover much more information about the stimulus and are thus advantageous^46–52^. In general, the amount of information extracted by the algorithm was an upper-bound. However, it is unclear how such weights can be assigned physiologically. Possible biological implementations of neural decoding with weighted sums have been investigated in previous studies^53,54^. For example, a model of population decoding with weights determined by the activity levels of upstream neurons can capture the experimentally observed behaviours^53^. In the electrosensory system, midbrain neurons of *torus semicircularis* in general integrate synaptic inputs from both ON- and OFF-type ELL pyramidal cells although the relative proportion varies greatly across individual neurons^55^. While a recent study showing that some midbrain neurons can reliably discriminate between different chirp stimulus waveforms provides support for the hypothesis that TS neurons respond to a weighted sum of ELL inputs^56^, further investigation is needed to fully test this hypothesis, and, if true, determine how the weights are assigned.

Our results show that ELL pyramidal cell populations can discriminate between chirps occurring at different phases of the beat. This is consistent with previous results showing good discriminability in peripheral electroreceptor afferents^57^ as these faithfully follow the detailed time course of the chirp stimulus^24,58^. Our results show that considering correlations between ELL pyramidal neuron activity can improve discriminability in the unequal-weighted case and we note that previous studies have shown other types of synergistic neural codes based on synchrony in both afferents^23,59^ and ELL pyramidal cells^60^. While behavioral studies have shown that fish can detect chirps with different attributes^24,58,61^, whether fish can discriminate between different chirp stimulus waveforms remains unknown as the behavioral responses were mostly invariant (i.e., the same) when varying chirp attributes such as amplitude, duration, and the phase of the beat at which the chirp occurs at^24,58^.

It is also important to note that our study focused on natural electrocommunication signals termed “small chirps” that tend to occur on top of low frequency beats^19,62^. There are other types of electrocommunication signals with different characteristics, e.g. “big chirps” that instead tend to occur on top of high frequency beats^19^. Interestingly, recent studies have shown that small chirps can also occur on top of high frequency beats^15^. Moreover, a previous study that considered population coding of both small and big chirps but did not consider the effects of noise correlations has found results qualitatively similar to our own when varying the timescale of encoding^33^. Further studies are needed in order to understand how correlations influence coding of big chirps as well as small chirps occurring on top of higher frequency beats by ELL pyramidal cell populations. Moreover, future studies should consider other behaviorally relevant stimulus classes (e.g., prey). We also note that the stimulation protocol using two electrodes on each side of the animal gives rise to stimulation patterns that are more homogeneous than those typically encountered during social interactions^63^. Future studies should take into account such patterns of stimulation when studying sensory processing by neural populations.

### Implications for other systems

Previous studies have shown that the electrosensory system processes information similarly to other sensory systems (e.g. contrast coding^64^, sensory adaptation^65^). Sensory processing of natural communication stimuli has been widely studied in other animals (e.g. songbirds^66,67^, grasshoppers^68,69^, the grassfrog^70^). We note that there are also similarities between the electrosensory system and other systems in terms of sensory processing of communication stimuli: for example, the midbrain *torus semicircularis* in the grassfrog contains neurons that selectively respond to natural mating calls^70^, while in the *torus semicircularis* of weakly electric fish *A. leptorhynchus*, neurons selectively respond to chirps were also found^24^. Therefore, we predict that our results are applicable to population coding of communication stimuli in other systems.

Our results further demonstrated the ON–OFF asymmetry of ELL pyramidal cells in terms of chirp discrimination. Previous studies showed symmetry between ON and OFF pyramidal cells in terms of their responses to different chirps (i.e., ON cells increase their firing rates while OFF cells decrease their firing rates in response to increases in stimulus amplitude)^24,64^. While the chirp stimuli we delivered contained equally phases that ON cells prefer and those that OFF cells prefer, ON cells still perform much better than OFF cells in discriminating different chirps, which is in agreement with previous studies^25^. Since we only used chirps with four different phases, future studies of chirp stimuli with more phases used can be done to further confirm an asymmetry in coding of chirp stimuli by ON and OFF type cells. ON and OFF type cells are found in other sensory modalities (e.g. visual^71,72^, auditory^73^, olfactory^74^). Other types of ON–OFF asymmetries have also been found previously in the visual system^75–78^. Our results thus add further evidence supporting the hypothesis that ON–OFF asymmetries are general property across different sensory modalities.

Methodologically, we used an evolutionary algorithm that runs iteratively to find weights that maximize discrimination performance, as was done recently for midbrain neurons^56^. The same algorithm was used previously to optimize model parameters^79^. The algorithm takes both spike timing and firing rate into account, therefore extracts information in not only the spike counts but the structures of spike trains. This algorithm can be easily adapted to analyze activities of neurons in other systems and help determine the upper-bound of information that the spiking activities of neurons can carry. We note that a similar approach was also used to optimize weights to maximize discriminability^45^.

## Materials and methods

### Animals

The South American wave-type weakly electric fish *Apteronotus leptorhynchus* (N = 2) was used in this study. Animals were purchased from tropical fish suppliers and were housed in groups (2–10) at controlled water temperatures (26–29 °C) and conductivities (300–800 µS cm^−1^) according to published guidelines^80^. All animal procedures were approved by McGill University’s animal care committee and were conducted according to the ARRIVE guidelines.

### Surgery and recording

Surgical procedures have been described in details previously^38^. Briefly, animals were immobilized by injection of 0.1–0.5 mg of tubocurarine (Sigma) intramuscularly. The animals were then transferred to an experimental tank (30 cm × 30 cm × 10 cm) containing water from the animal’s home tank and respirated by a mouth tube providing constant flow of oxygenated water at a flow rate of 10 mL min^−1^. Before surgery, the animal’s head was locally anesthetized with lidocaine ointment (5%; AstraZeneca, Mississauga, ON, Canada). Craniotomy (a ~ 5 mm^2^ window) was performed to partially expose the hindbrain. Neuropixel probes (Imec inc., Leuven, Belgium) were inserted into the brain along the rostral–caudal axis and a 45° angle with respect to the sagittal plane at transverse slice T-4 of the brain atlas (see^81^) laterally near the praeeminentialis efferent tract (labeled “tP-Cb” on the atlas), and the tip moved 1500 μm into the brain as measured from the surface. We waited at least one hour after probe insertion before starting recordings to allow brain tissue to settle following probe insertion and to improve recording stability. Accounting for the fact that the first recording site is located 175 μm away from the tip along the probe shaft, as well as the fact that recordings were typically obtained on recording sites ranging between 13 and 97, this gives approximate recording between 355 and 1195 μm from the brain surface along, which are within the range reported from a previous study where location within LS was confirmed by histological post-processing^82,83^. Thus, based on probe geometry, anatomy^81^, and our experience recording from ELL pyramidal cells^58,65,82,84,85^, it is likely that most of our recordings were from LS. However, we cannot reject the hypothesis that some of our recordings were from the centrolateral segment. The distance between recorded units was computed as the physical distance between the recording sites on which the spikes shapes of both units displayed the largest amplitude, which is approximate. However, since a given unit was most often recorded from the nearest neighbours to the primary recording site, the error is at most 40 μm based on probe geometry. We note that this is much smaller than the range of distances over which recordings were obtained.

### Stimulation

The electric organ discharge (EOD) of *A. leptorhynchus* is neurogenic, and therefore is not affected by injection of curare. Stimuli consisted of amplitude modulations (AM) of the animal’s own EOD were produced by triggering a function generator to emit one cycle of a sine wave for each zero crossing of the EOD as done previously^86^. The frequency of the emitted sine wave was set slightly higher (30 Hz) than that of the EOD, which allowed the output of the function generator to be synchronized with the EOD. The emitted sine wave was subsequently multiplied with the desired AM waveform (MT3 multiplier; Tucker Davis Technologies, Alachua, FL, USA), and the resulting signal was isolated from the ground (A395 linear stimulus isolator; World Precision Instruments, Sarasota, FL, USA). The isolated signal was then delivered through a pair of chloridized silver wire electrodes located 15 cm away from the animal on each side of the recording tank perpendicular to the fish’s rostro-caudal axis. In this study, a 5 Hz beat frequency and 14 ms chirp duration were used. Chirps were generated with different attributes by systematically varying the excursion frequency (30, 60, 90 and 120 Hz) and the phase (0, 90, 180 and 270°) of the underlying beat cycle at which the chirp occurs. As such, a total of 16 chirps were used (4 different chirp amplitudes, 4 different chirp phases). Parameter ranges were chosen to contain those observed in previous studies^17,87^. To measure the stimulus intensity, a dipole was placed near the animal's skin. Stimulus intensity was adjusted to produce changes in EOD amplitude that were ~ 20% of the baseline level, as done previously^24,88^. Each type of chirp stimulus was presented 40 times (i.e., 40 trials).

### Data analysis

Spike times for each individual neuron were sorted using Kilosort and manually curated using Phy 2. The spike times were converted into binary sequences *X*_*i*_*(t)* sampled at 2 kHz (i.e., 1 if a spike occurred during a given binwidth of 0.5 ms and 0 otherwise). Neurons were classified into either ON- or OFF-type based on spike-triggered average (STA) of a low-pass filtered (0–120 Hz) noise stimulus as done previously^89^. The strength of the neural response was quantified by the STA amplitude (i.e., the distance between the maximum and minimum values)^85^.

We quantified correlations between neuronal activities using spike count sequences *N*_*i*_ that were obtained from each spike train by counting the number of spikes occurring during 4 successive and non-overlapping 10 ms time windows that were always aligned with respect to 8 ms after the onset of the chirp stimulus in order to account for transmission delays. We then computed the correlation coefficient between pairs of spike count sequences using Pearson’s correlation coefficient:

1 $${r}_{ij}= \frac{\langle Cov({N}_{i}{, N}_{j})\rangle }{\sqrt{\langle Var({N}_{i})\rangle \langle Var({N}_{j})\rangle }}$$

where < *…* > represents an average over trials (i.e., each presentation of a given chirp stimulus is one trial). To compute signal correlations, spike count sequences were first randomly permuted based on the order of trials to obtain shuffled spike counts. Signal correlations were then computed on the shuffled spike counts using Eq. (1) and were averaged over 50 independent realizations of the shuffling procedure. Noise correlations were computed as the correlation coefficient between the spike count residual sequences, which were obtained by averaging over trials and subtracting the mean spike count sequence from the spike counts for each trial^56^. Thus, correlations were computed for each individual chirp stimulus.

For correlations at the population level, we divided the entire population into two subpopulations through partial sums: we summed the binary sequences *X*_*i*_*(t)* of 50% of the neurons in the entire population to form the first subpopulation and then the activities of the other 50% of the neurons to form the second subpopulation. Correlations were then computed as described above and error bands for signal and noise correlations were generated for 300 bootstrap samples of partial sums.

The single neuron PSTHs *R*_*i*_*(t)* were calculated by low-pass filtering the binary sequences *X*_*i*_*(t)* with a 10 ms boxcar window. The population PSTHs were obtained by summing the single neuron PSTHs *R*_*i*_*(t)* with either equal weights or unequal weights obtained through an evolutionary algorithm (described below):

2 $$equal-weight\;PSTH=\sum_{i=1}^{N}{R}_{i}\left(t\right)$$

3 $$unequal-weight\;PSTH= \sum_{i=1}^{N}{{w}_{i}R}_{i}\left(t\right)$$

where *w*_*i*_ is the weight of neuron *i*. We note that, as the weights can be negative, the population PSTH obtained using unequal weights can also be negative. For each stimulus, the population PSTH of each trial was then normalized by the maximal value of that trial. The mean and standard deviation of the normalized population PSTHs across different trials were then obtained.

To quantify the similarity of mean responses of the population to different stimuli, we computed the inter-response distance^24,79^:

4 $$D(x,y)=\frac{\sqrt{{<\left(x-y\right)}^{2}>}}{\mathrm{max}\left[\frac{\mathrm{max}\left(x\right)-\mathrm{min}\left(x\right)}{\sqrt{2}},\frac{\mathrm{max}\left(y\right)-\mathrm{min}\left(y\right)}{\sqrt{2}}\right]}$$

where x and y are means of normalized population PSTHs across different trials of two different stimuli, < … > denotes an average over an evaluation window of 40 ms after chirp onset. For each stimulus, we calculated the inter-response distance of the stimulus to the rest of the stimuli individually, and then took the average to obtain the averaged distance to other stimuli for this stimulus. For boxplots in Fig. 6c, the interquartile range (Q3 = 0.25, Q4 = 0.75) was taken to rule out stimuli whose averaged distances to other stimuli are either overly high or low, which hinder our comparisons.

To quantify the response variability of the population activities, we averaged the standard deviation of responses across different trials over all stimuli:

5 $$RV=\sum_{i=1}^{n}\frac{\sigma \left({k}_{n}\right)}{n}$$

where σ(k_n_) is the standard deviation of normalized population PSTHs across different trials of each stimulus and n is the number of stimuli. The response variability at each time point was normalized by the maximal value of variability across the entire evaluation time window. For boxplots in Fig. 6d, the interquartile range (Q3 = 0.25, Q4 = 0.75) was taken to rule out times at which variability values are either overly high or low.

### Classifier

We used a classifier to quantify the performance of ELL pyramidal cells at stimulus discrimination. We combined activities of individual neurons using either weighted or un-weighted sums for each chirp stimulus. For each chirp stimulus, the averaged population activity of all trials was chosen as a template. Next, each combined response was assigned as being generated by the stimulus that gave rise to a given template based on whether the distance between the combined response and the template was minimum. We thus constructed a “confusion matrix” whose element (*i,j*) gives the probability that a response was assigned as being generated by stimulus *j* given that it was actually generated by stimulus *i*^26,89,90^. The diagonal elements of this matrix are the probabilities that a stimulus was correctly assigned, whereas non-zero off-diagonal elements indicate misclassification. For each confusion matrix we computed the discrimination performance by averaging over the diagonal elements, as done previously^26,56,89^. The discrimination performance can thus vary between 0 (no discrimination) and 1 (perfect discrimination). Note that the chance level for discrimination performance was 0.0625 (that is, 1/16) because we used a total of 16 different chirp stimuli. The distance between combined neuron activities was computed using the van Rossum metric^32^. First, the combined neural activities were convolved with a decaying exponential kernel with time constant *τ*:

4 $$f\left(t\right)= {\sum }_{i=1}^{M}H(t-{t}_{i}){e}^{\frac{-(t-{t}_{i})}{\tau }}$$

where *t*_*i*_ is the *i*th spike time, *M* is the total number of spikes and *H(t)* is the Heaviside step function (*H(x)* = 0 if x < 0 and *H(x)* = 1 if x >  = 0). The distance was then computed as the Euclidian distance between convolved combined neural activities *f*_*Rj*_ and *f*_*Rk*_:

5 $${D({f}_{{R}_{j}},{f}_{{R}_{k}})}_{\tau }= \sqrt{\frac{1}{\tau }\int {\left[{f}_{{R}_{j}}- {f}_{{R}_{k}}\right]}^{2}dt}$$

We varied *τ* between 1 and 100 ms to evaluate the effects of precise spike timing on classification. When *τ* is small, the metric takes into account spike timing whereas, when *τ* is larger, the metric takes into account slower changes in firing rate. If not specified otherwise, *τ* = 3 ms was used.

### Evolutionary algorithm

In order to determine whether performing a weighted sum of neural response gave rise to better classification than an equal-weight sum, we trained an evolutionary algorithm (EA) using the population responses on a randomly selected 60% of trials for each chirp stimulus as a training dataset. We then measured the classification accuracy of the trained classifier on the entire dataset. We chose the recording session that contained the greatest number of neurons recorded simultaneously (n = 21).

Specifically, each neuron was assigned a weight *w*_*i*_ which varies between − 2 and 2 and the goal was to choose a set of weights that maximizes the performance of the classification algorithm described above. The EA is described in detail in a previous studies by our group^56,79^. Specifically, a set of weight vectors (i.e., “agents”) is allowed to evolve by minimizing a fitness function *F*_*fit*_ over a series of iterations (i.e., “generations”). In keeping with the notation used in previous studies^79^, we denote ${X}_{k}^{r}\left(i\right)$ as parameter *i* for agent *r* of generation *k*. First, the population of *K* individuals is randomly initialized with weight values that are uniformly distributed with zero mean and restrained within [− 2 2]. For each individual at every generation, a new individual is constructed by “differentiation”: the *r*th new parameter vector ${X}_{k, trial}^{r}$ is built by combining three other individuals ${X}_{k}^{{r}_{1}}$, ${X}_{k}^{{r}_{2}}$, and ${X}_{k}^{{r}_{3}}$, where r_1_ ≠ r_2_ ≠ r_3_:

6 $${X}_{k,trial}^{r}= {X}_{k}^{{r}_{1}}+\left({X}_{k}^{{r}_{2}}-{X}_{k}^{{r}_{3}}\right)F, \forall r= 1,\dots ,N$$

where the differential weight *F* = 0.5, and the three individuals are chosen based on a probability distribution that is preferentially weighted for more fit (i.e., lower fitness score) individuals:

7 $${p}_{k}^{{r}_{1}}=\lambda exp\left(\frac{{-F}_{fit}\left({X}_{k}^{{r}_{i}}\right)}{\underset{\forall j}{\mathrm{max}}({1-F}_{fit}\left({X}_{k}^{j}\right))}\right), \forall {r}_{i}=1,\dots ,N$$

where *λ* is a normalization constant such that the sum of probability values is equal to one. Random mutations are then performed as follows:

8 $$ X_{{mut}}^{r} (i) = \left\{ {\begin{array}{*{20}l}    {X_{{k,trial}}^{r} \left( i \right),} \hfill & {if\;u < CR} \hfill  \\    {X_{k}^{r} (i),} \hfill & {otherwise} \hfill  \\   \end{array} } \right.,\forall r = 1, \ldots ,N;i = 1, \ldots ,D $$

where *u* is a random variable generated from a uniform distribution *U*(0,1) and with crossover probability *CR* = 0.9. Selection is finally performed to produce the next generation via:

9 $$ X_{{k + 1}}^{r}  = \left\{ {\begin{array}{*{20}l}    {X_{{mut}}^{r} ,} \hfill & {if\;F_{{fit}} \left( {X_{{mut}}^{r} } \right) < F_{{fit}} \left( {X_{k}^{r} } \right)} \hfill  \\    {X_{k}^{r} ,} \hfill & {otherwise} \hfill  \\   \end{array} } \right.,\forall r = 1, \ldots ,N $$

In this study, the fitness function for a given individual was defined as:

10 $${F}_{fit}\left({X}_{k}^{r}\right)= {1-DP}_{{X}_{k}^{r}}$$

where ${DP}_{{X}_{k}^{r}}$ is the discrimination performance estimated by computing the precision of events (i.e., spikes) of our neuronal population in response to our set of 16 chirp stimuli. The EA was terminated if the change in population discrimination performance was less than 0.0001 in 10 consecutive iterations. The algorithm was repeated 30 times, and each time a different set of weights was obtained because of different initial conditions and the randomness in generating new individual and mutations. The weights were normalized so that the sum of weights of all neurons equals to 1. The weights that gave rise to the best performance out of the 30 runs were used for Figs. 6 and 7. As mentioned above, this methodology is the same as that used previously for midbrain neurons^56^, which allows for a direct comparison between these previous results and those obtained in the current study for hindbrain neurons. In general, we found significant correlations between weight magnitude and STA amplitude to noise stimulus for ON (r = 0.92, p = 6.4 × 10^–7^) and OFF cells (r = 0.95, p = 0.013).
